# Functions of OsDof25 in regulation of *OsC4PPDK*

**DOI:** 10.1007/s11103-015-0357-3

**Published:** 2015-09-03

**Authors:** Y. Zhang, N. I. Verhoeff, Z. Chen, S. Chen, Mei Wang, Zhen Zhu, P. B. F. Ouwerkerk

**Affiliations:** Department of Molecular and Developmental Genetics, Institute of Biology (IBL), Leiden University, P.O. Box 9505, 2300 RA Leiden, The Netherlands; State Key Laboratory of Plant Genomics and National Center for Plant Gene Research, Institute of Genetics and Developmental Biology, Chinese Academy of Sciences, No. 1 West Beichen Road, Chaoyang District, Beijing, 100101 China; Graduate School of the Chinese Academy of Sciences, Beijing, 100049 China; Biotechnology Research Institute, Fujian Academy of Agricultural Sciences, Wusi Rd 247, Fuzhou, 350003 Fujian China; SU BioMedicine/TNO Quality of Life, Zernikedreef 9, P.O. Box 2215, 2301 CE Leiden, The Netherlands

**Keywords:** Dof, *OsC4PPDK*, Transcription factor, Rice

## Abstract

Relative little is known about the functions of the so-called Dof zinc factors in plants. Here we report on the analysis of OsDof25 and show a function in regulation of the important C4 photosynthesis gene, *OsC4PPDK* in rice. Over-expression of *OsDof25* enhanced the expression of *OsC4PPDK* in transient expression experiments by binding in a specific way to a conserved Dof binding site which was confirmed by yeast and in vitro binding studies. Expression studies using promoter GUS plants as well as qPCR experiments showed that *OsDof25* expressed in different tissues including both photosynthetic and non-photosynthetic organs and that expression of OsDof25 was partially overlapping with the *OsC4PPDK* gene. Conclusive evidence for a role of OsDof25 in regulation of *C4PPDK* came from loss-of-function and gain-of-function experiments with transgenic rice, which showed that down-regulation or over-expression of *OsDof25* correlated with *OsC4PPDK* expression and that OsDof25 has functions as transcriptional activator.

**Electronic supplementary material:**

The online version of this article (doi:10.1007/s11103-015-0357-3) contains supplementary material, which is available to authorized users.

## Introduction

Rice (*Oryza sativa* L.) is one of the most important food crops in the world because it feeds more than half of global population and is daily carbohydrate source in many countries. Traditional breeding only results in an annual yield increase of about 1 % whereas this should be higher to keep in pace with the increase in demand. Much is expected from molecular breeding as a tool to combine the best genes and alleles in novel plant types but to enable this we need new information about which genes and alleles are responsible for which traits. To enable this process, many national and international genetics and genomics projects on rice were initiated in the last decade and as results the genomes of indica and japonica have been completely or partially sequenced (Matsumoto et al. 2005), a large number of T-DNA insertion or transposon-based tagged mutants have been produced (Hirochika et al. 2004; Jung and An 2013; Priya and Jain 2013), genome wide expression profiles have been obtained by using microarray and SAGE (Bao et al. 2005; Li et al. 2006). All these approaches have provided very useful information and resources enabling functional genomics with rice. However, the function of the majority of rice genes has not been experimentally demonstrated (Zhang et al. 2006). In particular knowledge on functions of transcription factors and other regulatory proteins could be relevant for improvement of rice since some of them are related to important traits in cereals. Examples of such transcription factors are Opaque2 from maize that is a regulator of seed-storage protein deposition and TB1 which plays a major role in tiller number and apical dominance in maize (Hwang et al. 2004). In rice, MOC1 was shown to be important in determining tiller number (Li et al. 2003) and OsSPL14 was shown to be involved in tiller number and grain per panicle number (Luo et al. 2012).

Transcription factors play key roles in regulating gene expression at the transcriptional level. According to the rice transcription factors database, there are at least 63 transcription factor families in the rice genome (Gao et al. 2006). One of them is the Dof gene family (for reviews see Yanagisawa 2002; Noguero et al. 2013) which has important functions in responses to plant hormones such as gibberellin (Washio 2001; Mena et al. 2002) and auxin (DePaolis et al. 1996; Kisu et al. 1998), stress responses (Zhang et al. 1995), flowering time (Li et al. 2009; Corrales et al. 2014), tissue specific expression (Yanagisawa 1998; Plesch et al. 2001) and photosynthesis (Yanagisawa and Sheen 1998; Yanagisawa 2000). Dof transcription factors have one copy of the Dof zinc finger domain (hence the name of DNA binding with only one finger), which normally resides in the N-terminal region, but the sequences outside the Dof domains are very diverse (Riechmann and Ratcliffe 2000; Yanagisawa 1998, 2002). Most of the Dof domain proteins recognise an AAAG motif or the revise complement CTTT as core sequence element in DNA binding assays in vitro (Yanagisawa 2002), except for AOBP, a protein from pumpkin, which recognises an AGTA repeat as core binding sequence (Kisu et al. 1998).

*ZmDof1* was found to be associated with expression of multiple genes involved in carbon fixation in maize (Yanagisawa 2000), and was shown to be able to activate expression of the *OsC4PPDK* promoter by binding to the AAAG motif (Yanagisawa 2000). *OsC4PPDK* (pyruvate orthophosphate dikinase) catalyses the conversion of pyruvate into phosphoenolpyruvate (Edwards et al. 1985). This is one of the key steps in the C4 photosynthesis pathway, since PPDK can regenerate the primary CO_2_ acceptor phosphoenolpyruvate (PEP) which is the substrate for an important step in carbon fixation in mesophyll cell chloroplasts (Chollet et al. 1996). Since carbon fixation is the most important process underlying yield in cereals, we wanted to study whether Dof transcription factors can be used as a tool to modify *OsC4PPDK* expression. As a first step, we cloned *OsDof25* from rice which together with *OsDof24* is the closest homolog to *ZmDof1* on the protein level and investigated its function in more detail using loss-of-function and gain-of-function studies supported by in vitro binding studies. The results show novel insight in regulation of *OsC4PPDK* by OsDof25 and will ultimately contribute to understanding the regulation of C3 and C4 photosynthesis genes in rice.

## Materials and methods

### Sequence alignments and phylogeny analysis

The sequence of the ZmDof1 protein was obtained from GenBank ABF51012 (Yanagisawa 2001; Cavalar et al. 2007). All protein sequences of rice Dof transcription factors were downloaded from the rice transcription factor database (http://ricetfdb.bio.uni-potsdam.de/v2.1/) (Riano-Pachon et al. 2007). The amino acid sequences of ZmDof1 and the rice Dof proteins were aligned using ClustalX (2.0). The Neighbor-Joining algorithm implemented in MEG4 (Tamura et al. 2007) was used for the phylogenetic tree assay. Two hundred bootstrapped data sets were used to estimate the confidence of each clade tree.

### RNA isolation, RT-PCR, gene cloning and sequence analysis

Flag leaves harvested 10 DAF from rice hybrid Liangyou 2186 (*Oryza sativa* L. ssp. *indica*) were ground in liquid nitrogen and total RNA was extracted by using Trizol according to the manufacturer’s instructions (Invitrogen). Genomic DNA contaminants were removed from RNA samples by incubating with DNA-free™ (Ambion) at 37 °C for 30 min. First-strand cDNA was synthesised starting from 1 µg of total RNA with SuperScript III reverse transcriptase (Invitrogen) as described by the manufacturer.

The RT-PCR reactions were performed based on single-strand cDNA. The primer OsDof25-F1 and OsDof25-R1 (Table S3) were used to get the cDNA of *OsDof25*. PCR conditions were 5 min of initial denaturation at 98 °C, 36 cycles of denaturation at 98 °C for 30 s, annealing at 62 °C for 30 s and extension at 72 °C for 45 s, followed by a final extension step at 72 °C for 10 min. The PCR products were separated in a 1× TBE, 1 % agarose gel.

To confirm the identity of the amplified sequences, the PCR products were cloned in pCR-Blunt II-TOPO (Invitrogen) and sequenced commercially (BaseClear, Leiden, The Netherlands). The sequences of *OsDof25* were further analysed using DNAMAN.

### Construction of effector and reporter plasmids, transient transformation of rice protoplasts and GUS assays

An effector plasmid, Pro35S-OsDof25, was made by insertion of an *Xho*I–*Kpn*I fragment from pCR-blunt II-TOPO-OsDof25 into expression vector pRT100 (Töpfer et al. 1987). For the loss-of-function analysis, GUS reporter plasmids carrying different lengths of the *OsC4PPDK* promoter were generated by insertion of different PCR fragments of the *OsC4PPDK* promoter using the *Bam*HI and *Nco*I sites into vector pGusXX (Pasquali et al. 1994). For the gain-of-function analysis, wild type sequence and mutant fragments from −385 to −274 of *OsC4PPDK* promoter were obtained by PCR and then digested with *Not*I/*Spe*I and inserted into plasmid pGusXX-47 (Pasquali et al. 1994) which has a minimal TATA box containing fragment from the CaMV 35S promoter. Reporter plasmids ProPPDK-A::GUS, ProPPDK-B::GUS, ProPPDK-C::GUS, ProPPDK-D::GUS and ProPPDK-E::GUS (Fig. 6) represent the fragment containing wild type CTTT motif, mutant motifs GTTT, CATT, CTAT and CTTA respectively. The primers used to construct the reporter plasmids construction are listed in Table S3.

Starting material for the protoplast experiments were 2 weeks old seedlings. Seeds of rice cultivar Minghui 86 (*Oryza sativa* L. ssp. *indica*) were allowed to imbibe in water at darkness at room temperature for 3 days. Next, the seeds were sown in soil and grown at 26 °C, 80 % humidity and 12/12 dark/light period, with a light intensity (photosynthetically active radiation value) of 180 µmol/m^2^/s. Protoplast isolation and transfection was essentially performed as described by Chen et al. (2006). Cotransformations were performed with 4 µg reporter plasmids and 6 µg effector plasmids using a PEG-based transformation method. Co-transformations with empty overexpression vectors served as controls. After incubation in 1.5 ml W5 buffer in a dark room at 28 °C overnight, protoplasts were harvested and total protein were isolated and then frozen in liquid nitrogen. GUS activity assays were performed as described by Van der Fits and Memelink (1997) and protein concentrations were measured using the Bradford protein assay reagent (BioRad). Each experiment was performed at least three times and the relative GUS activities of duplicate samples were normalised for total protein.

### Recombinant OsDof25 protein expression and electrophoretic mobility shift assays (EMSA)

The fragment of *OsDof25* cDNA was fused in-frame with the GST sequence in expression vector pGEX-KG (Guan and Dixon 1991) by sub-cloning *Bam*HI–*Eco*RI fragments from construct Pro35::OsDof25. In order to extract recombinant protein, 5 ml of overnight cultures of BL21 (DE3 pLys) (Novagen) carrying pGEX-KG-OsDof25 plasmids were used to inoculate 500 ml LB medium containing 200 µg/ml carbenicillin and 50 µg/ml chloramphenicol, which was incubated at 37 °C to OD600 0.5. Next, protein synthesis was induced by the addition of solid IPTG to final concentration of 1 mM and cultures were incubated for 4 h at 29 °C. The harvested cells were suspended in 20 ml PBS and frozen in liquid nitrogen. After thawing pellets at 37 °C, the bacteria were lysed by sonication (eight times 10 s burst; 5 s pause between bursts), and centrifuged (at 18,000 rpm for 30 min at 4 °C), then the supernatant was filtered through a 0.45 µm membrane. Protein purification was performed using Poly-Prep Chromatography columns (Biorad 731-1550) containing 0.5 ml settled Glutathion-Sepharose 4B beads (Amersham Biosciences). Columns were first washed two times with 10 ml PBS before bacterial extract was passed through. After binding, columns were washing with 10 ml PBS and bound proteins were eluted in 2.5 ml (10 × 0.25 ml) glutathion elution buffer (100 mM glutathione, 500 mM Tris–HCL pH 8.0). Eluted protein was concentrated using Microcone centrifugal filter devices (Millipore) according to manufacturer’s instruction and the protein content was determined by the method of Bradford. The GST-OsDof25 fusion protein was stored at −80 °C in 10 % glycerol.

All EMSA reactions contained 100 ng poly-(dI-dC)-poly-(dI-dC) (Amersham-Pharmacia) and 1 ng of ^32^P end-labeled probe (~10^8^ cpm/µg) in nuclear extraction buffer (Green et al. 1987). Labeled probes were incubated with GST-OsDof25 proteins together at room temperature 30 min and then were loading on a native 4 % polyacrylamide (30:0.8) gel in 0.5× TBE while under current. Probes used in labeling originated from annealed oligonucleotides are listed in Table S2.

### DNA binding specificity of OsDof25 proteins in yeast one-hybrid assay

Fragments P3 and P6 from the *C4PPDK* promoter, containing the putative Dof binding motif CTTT and its mutant derivative CTAT, respectively, were obtained by annealing the primers listed in Table S2 and then cloned into yeast integrative vector pINT1-HIS3NB (GenBank Accession AY061966; Ouwerkerk and Meijer 2011) between *Not*I and *Spe*I sites. The resulting plasmids ProOsC4PPDK-WT::HIS3 and ProOsC4PPDK-MU::HIS3 were confirmed by sequencing. Next, the *HIS3* reporter-containing fragments were excised with *Sac*I–*Nco*I and introduced into yeast strain Y187 (*MATα*, *ura3*-*52*, *his3*-*Δ200*, *ade2*-*101*, *trp1*-*901*, *leu2*-*3*, *112*, *met*^−^, *gal4gal80*, *URA3:GAL1*_*UAS*_-*GAL1*_*TATA*_-*lacZ;* Clontech) (Meijer et al. 1998; Ouwerkerk and Meijer 2001, 2011) resulting in yeast strains Y187:ProOsC4PPDK-WT and Y187:ProOsC4PPDK-MU. YPO101 is a control strain used in yeast one-hybrid assays.

An *Eco*RI/*Bam*HI fragment from pRT100-OsDof25 containing the *OsDof25* ORF was inserted into pACTIIa (Meijer et al. 1997) to generate plasmid pACTIIa-OsDof25, which has a fusion between OsDof25 and the GAL4 AD in order to carry out DNA binding assays in a yeast one-hybrid system. For this, plasmid pACTIIa-OsDof25 was introduced into yeast strains Y187:ProOsC4PPDK-WT, Y187:ProOsC4PPDK-MU and YPO101. The empty plasmid pACTIIa was transformed into the same strains as negative control.

Yeast transformations were performed as earlier described (Ouwerkerk and Meijer 2001). The transformed yeast cells were plated on CM/−Leu-His+Met+Ade+Trp medium and incubated at 30 °C and usually after 4 or 5 days colonies appeared. Next, the resulting colonies were streaked on CM/−Leu+His+Met+Ade+Trp medium including different concentrations of 3-AT. The plates were incubated at 30 °C for 1 week before scoring. All handlings with yeast were as described earlier (Meijer et al. 2000; Ouwerkerk and Meijer 2001, 2011).

### Sub-cellular localisation of OsDof25 protein

The ORF of *OsDof25* was amplified with primers OsDof25-F2 and OsDof25-R2 (Table S3) from the Topo vector. The PCR product was fused in frame to the N-terminus of the green fluorescent protein (GFP) gene to generate plasmid Pro35S::OsDof25::GFP. Vector pTH2 (Pro35S::GFP) was used as a control (Chiu et al. 1996). In this plasmid the S65T sGFP gene is driven by the CaMV 35S promoter and no specific localisation signals are present. Protoplasts isolated from 2 weeks old rice seedlings were transiently transformed using a PEG-mediated method (Chen et al. 2006). The transformed protoplasts were incubated at 28 °C overnight in K3 buffer and then checked for expression using a then observed using a Nikon Eclipse Ci fluorescence microscope. Excitation and emission filters for GFP detection were Ex470-490/DM505/BA520-560.

### Binary vector construction and plant transformation

To generate a *OsDof25* (LOC_Os09g29960) promoter GUS fusion construct, a 2368 bp DNA sequence upstream of the predicted *OsDof25* translation start site was amplified by PCR from genomic DNA isolated from Minghui 86 using Phusion polymerase (Invitrogen) and primers ProOsDof25-F and ProOsDof25-R (Table S3). The PCR fragment was subsequently cloned into vector pCR2.1 Topo (Invitrogen) for sequence analysis and then inserted into vector pCAMBIA-1391Z (GenBank Accession AF234312) resulting in a fusion with the GUS reporter gene. An *OsC4PPDK* promoter GUS fusion construct (ProOsC4PPDK::GUS) was generated by insertion of a 2572 bp *Eco*RI–*Nco*I fragment *OsC4PPDK* promoter, into pCAMBIA-1391Z. The resulting constructs were used for rice transformation.

To construct an *OsDof25* vector for overexpression, the full length coding sequence was amplified by PCR with primers OsDof25-F5 and OsDof25-R5 (Table S3) and then inserted as *Nco*I/*Bam*HI fragment between the *GOS2* promoter and *nos* terminator in binary vector pCAMBIA-1300intC (GenBank Accession AF294978). The resulting construct was used for rice transformation. The expression of *OsDof25* was down-regulated by an RNAi approach based on generating transgenic plants equipped with a pHANNIBAL silencing vector (Wesley et al. 2001). Specific *OsDof25* sense and anti-sense sequences were obtained by PCR and then inserted into pHANNIBAL in a two-step cloning procedure. First, the anti-sense fragment of *OsDof25* was generated using primers OsDof25-F6 and OsDof25-R6 (Table S3), then digested by *Cla*I/*Bam*HI, and inserted into pHANNIBAL vector between the same sites. Second, the sense fragment of *OsDof25* was generated using primers OsDof25-F7 and OsDof25-R7 (Table S3) and then digested with *Xho*I/*Kpn*I, and inserted into pHANNIBAL already carrying the anti-sense fragment. The resulting plasmid was digested with *Sal*I/*Spe*I and the generating fragments was inserted into pCAMBIA-1300intC between *Sal*I and *Xba*I. The resulting binary vector was used in rice transformation.

The *OsC4PPDK* promoter (LOC_Os05g33570) was obtained by PCR on genomic DNA isolated from indica rice cultivars Minghui 86 and SE21S using primers ProOsC4PPDK-F and ProOsC4PPDK-R (Table S3). To confirm the identity of the amplified sequences, PCR products were cloned in pCR-Blunt II-TOPO vector (Invitrogen) and sequenced by BaseClear (Leiden, The Netherlands).

Transformation of the *japonica* rice cultivar Zhonghua 11 with the above descried binary vector constructs was performed as described by Scarpella (Scarpella et al. 2000) except that *A. tumefaciens* LBA4404 was used for all rice transformations. Prior to growth in the greenhouse, transgenic seedlings were selected on a half-strength Murashige–Skoog medium supplied with 0.7 % type I agarose (Sigma) and 25 µg/ml hygromycin. By using Southern blotting with *hpt* as a probe, we identified single copy T-DNA lines.

### Detection of GUS expression in transgenic rice, cytological techniques and microscopy

Histochemical detection of GUS activity, cytological techniques and microscopy were performed as described previous (Scarpella et al. 2000). Samples were viewed using a Leica MZ12 stereo microscope or a Leitz Diaplan microscope with bright-field optics settings and images were acquired with a Sony 3CCD Digital Photo Camera DKC-5000.

### Expression assays of OsDof25 and OsC4PPDK using qPCR

To study the expression profile of *OsDof25* and *OsC4PPDK*, a qPCR analysis was done on a collection of nine tissues, including 2-week old seedlings and eight different tissue samples from mature Minghui 86 plants, including stems, roots, sheath, flag leaves, penultimate leaves and panicles at 10 DAF (days after flowering). For the expression analysis of *OsDof25* and *OsC4PPDK* in over-expression and RNAi transgenic lines, total RNA was isolated from 10 DAF flag leaves using Trizol kit (Invitrogen). RT reactions were performed with SuperScript™ II reverse transcriptase (Invitrogen) following the manufacturer’s instructions. Reactions were performed in an optical 96-well plate with an ABI PRISM^®^ 7500 Real-time PCR System (Applied Biosystems) by using SYBR^®^ Green to monitor dsDNA synthesis. All reactions contained 12.5 µl 2× SYBR^®^ Green Master Mix Reagent (Applied Biosystems), 2.0 ng cDNA and 10 pmol of each gene-specific primer in a final volume of 20 µl. Thermal cycling was as follows: 50 °C for 2 min; 95 °C for 10 min; 50 cycles of 95 °C for 10 s, 60 °C for 30 s, 72 °C for 30 s. Relative expression levels of reporter and target genes were calculated using the $2^{{ - {\varDelta \varDelta }C_{\text{T}} }}$ method (Livak and Schmittgen 2001) using rice *Actin1* and *Ubiquitin* as internal control. An overview of primers used in qPCR is shown in Table S4.

### Accession numbers

Sequence data from this article can be found in the EMBL/GenBank databases with accession codes KC996732, KC996733 and KP772258 for the *OsC4PPDK* promoter, the *OsDof25* cDNA clone and the *OsDof25* promoter, respectively.

## Results

### Phylogenetic analysis of the japonica rice Dof transcription factor family and identification of *OsDof24* and *OsDof25* as closest homologs of *ZmDof1*

**Fig. 1:**
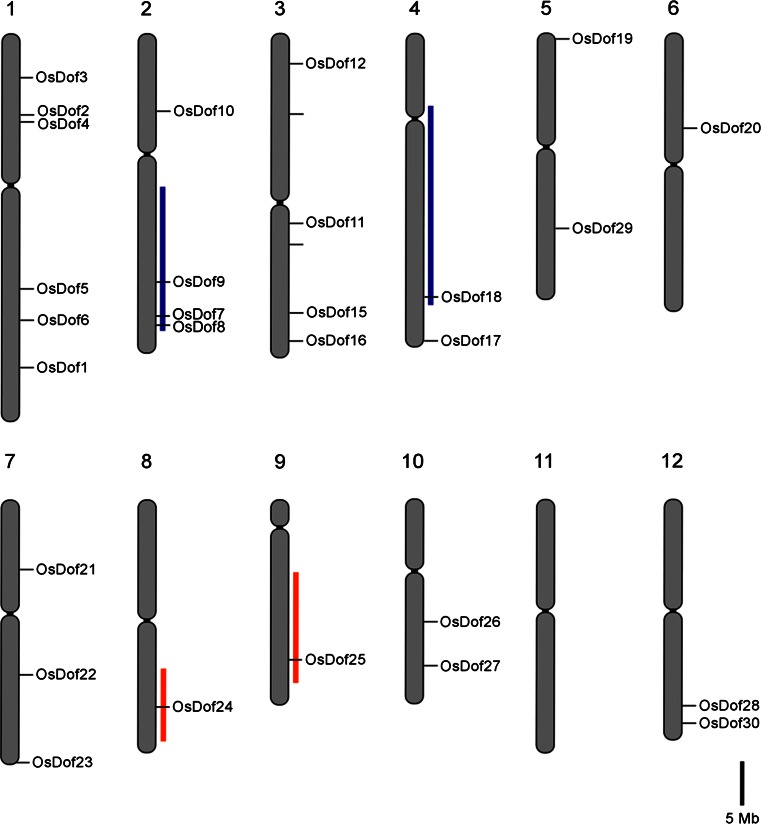
Chromosomal distribution of all Dof transcription factors on the 12 rice chromosomes. Duplicated regions basing on the whole-genome duplication (Yu et al. 2005) are indicated with different colours. *Scale bar* is 5 Mb

A bioinformatics analysis of the japonica rice genome identified 30 Dof transcription factor genes (*Oryza sativa*) (Lijavetzky et al. 2003). All Dof transcription factor genes have been assigned to a chromosomal map position (Fig. 1; Table S1) which is based on the TIGR database (http://plntfdb.bio.uni-potsdam.de/v3.0/) (Ouyang et al. 2007). To determine if there are any paralogous gene pairs we checked their chromosomal locations in relation to the history of genome duplications (Yu et al. 2005). The chromosomal map presented in Fig. 1 shows that the rice Dof genes are not generally clustered and that the pair *OsDof7*, −*8*, −*9/*−*18* and the pair *OsDof24/*−*25* are very likely paralogues because they are located in duplicated regions on chromosomes 2/4 and 8/9 respectively.

**Fig. 2:**
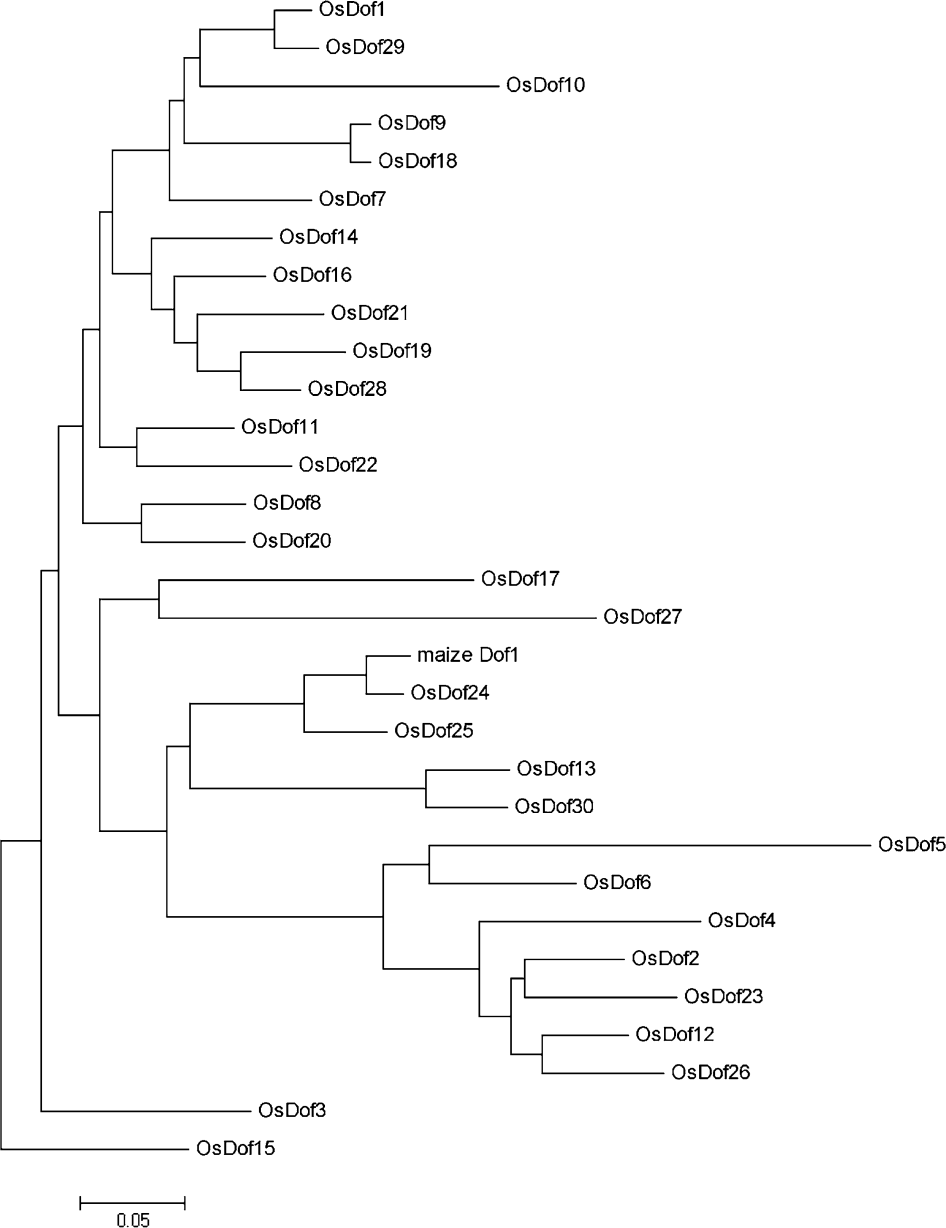
Phylogenetic tree analysis of *ZmDof1* and rice Dof transcription factors proteins. The amino acid residues of the entire Dof transcription factor family were aligned using ClustalX and the tree was created using MEGA4 software

Alignments of the amino acid sequences of the Dof domains of all 30 Dof proteins, which were downloaded from the transcription factor database (Riano-Pachon et al. 2007), showed that the Dof domains were highly conserved but that outside of the Dof domain there is little conservation (Yanagisawa 2002). Next, we performed a phylogenetic analysis with the rice Dof family and ZmDof1. As shown in Fig. 2, there are two Dof proteins from rice, OsDof24 and OsDof25, in the same clade with ZmDof1. Given the role of ZmDof1 in regulation of the photosynthesis genes *PEPC* and *C4PPDK*, it may very well be that OsDof25 is also involved in regulation of *OsC4PPDK*.

In order to further study the functions of *OsDof25*, this gene was cloned from the Chinese super-hybrid rice combination Liangyou 2186 (GenBank Accession KC996733). Sequence analysis showed that there are two extra serine residues on position 97 compared with the sequence downloaded from the plant transcription factor database (http://plntfdb.bio.uni-potsdam.de/v3.0/). Likely these two serines do not have an serious effect on functionality of the Dof domain of OsDof25 since they are outside this domain (data not shown).

### OsDof 25 is an activator of the *OsC4PPDK* promoter

**Fig. 3:**
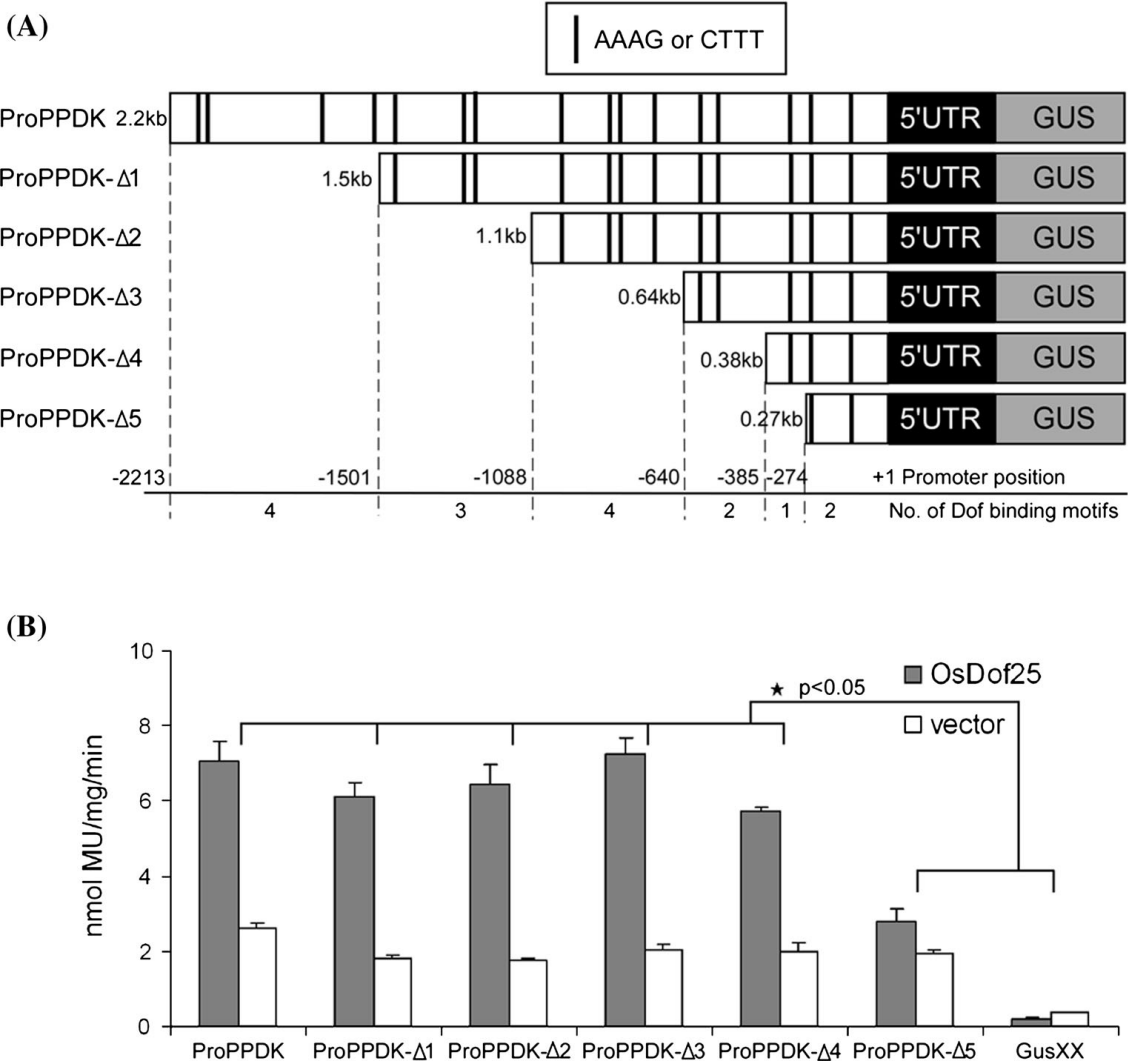
Loss-of-function analysis of the *OsC4PPDK* promoter in rice protoplasts. **a** Schematic representation of truncated promoter-GUS constructs that were used in transient expression assays with rice protoplasts. Promoter lengths are indicated on the *left*. Negative numerals indicate the nucleotide position relative to the transcriptional start site. Putative Dof binding sites, AAAG or CTTT, as predicted by the PLACE database are indicated by *black bars*. Numbers of the putative binding motif within truncated regions are indicated. **b** The effects of OsDof25 overexpression and the mapping of OsDof25-binding fragments on the *OsC4PPDK* promoter were tested using an overexpression construct Pro35S::OsDof25 which was co-transformed into rice protoplasts with a series of *OsC4PPDK* promoter GUS constructs. The empty effector plasmid pRT100 was used as a control. Relative GUS activities were normalised for total protein. GUS activities of co-transformation with Pro35S::OsDof25 are indicated in *black* and columns representing empty effector plasmids are *blank*. The *bar graphs* are based on the mean values of three independent transformations of each construct combination and *error bars* represent means and standard deviations (SDs) of biological replicates. The data were analysed using ANOVA followed by Bonferroni corrections. *Asterisks* indicate significant differences (*p* < 0.05) compared with the untransformed controls

To determine whether OsDof25 can regulate the expression of *OsC4PPDK* an experimental set-up was designed based on transient expression assays in rice protoplasts using OsDof25 effector constructs and *OsC4PPDK* promoter reporter constructs. For this, a 2.5 kb promoter fragment of *OsC4PPDK* was cloned from rice cultivar SE21S and Minghui86 (*Oryza sativa* L. ssp. *indica*) which are the parents of hybrid rice combination Liangyou 2186. Sequencing and alignments showed that there is no difference between the *OsC4PPDK* promoters from cultivars SE21, Minghui 86 (GenBank Accession KC996732) and Nipponbare. The reporter *ProPPDK::GUS* was constructed by introducing a 2.5 kb *OsC4PPDK* promoter fragment fused to the GUS reporter gene as a transcriptional fusion. Plasmid Pro35S::OsDof25, carrying the ORF of *OsDof25* under the control of the *CaMV 35S* promoter was used as effector. As shown in Fig. 3, a combination of this reporter and effector resulted in a doubled GUS activity compared to the empty effector plasmid pRT100. Thus the results strongly suggested that OsDof25 can activate expression of the *OsC4PPDK* promoter in vivo.

It has been reported that most of Dof transcription factors will recognise the sequence AAAG or its reverse complementary sequence, CTTT, as an essential DNA binding motif (Yanagisawa and Izui 1993; Yanagisawa and Schmidt 1999; Yanagisawa 2002). We used PLACE (Higo et al. 1998) to search for *cis*-acting elements, in the 2.5 kb *OsC4PPDK* promoter fragment, and as a result 16 putative binding motifs (AAAG or CTTT) were identified which are indicated in Fig. 3a. To further study the function of the putative Dof binding sites identified in the *PPDK* promoter, a loss-of-function experiment was designed based on a series of five deletion constructs (ProPPDK-∆1::GUS to ProPPDK-∆5::GUS) fused to the GUS reporter gene (Fig. 3a). As shown in Fig. 3b, only co-transfection with reporter ProPPDK-∆5::GUS and effector Pro35S::OsDof25 did not result in an increase of relative GUS activity, but co-transfection with the other reporters showed a similar increase of relative GUS activity compared to the full length *OsC4PPDK* promoter. The difference between ProPPDK-∆4::GUS and ProPPDK-∆5::GUS is that there is a 111 bp deletion from −385 to −274, which contains one putative Dof binding motif with the consensus sequence CTTT (Fig. 3a) which in principle could be binding site for the OsDof25 protein. Taken together, the results showed that OsDof25 can activate a series of *OsC4PPDK* promoter deletion constructs. Up to coordinate −385, deletion had little effect on activation by OsDof25. In turn, when a 111 bp fragment spanning from −385 to −274 bp containing a putative Dof binding motif was deleted, activation by OsDof25 decreased from twofold to only onefold. Although it seems that this particular region in the *OsC4PPDK* promoter is important in regulation we do not exclude that there are important elements further upstream in the promoter which also contribute to expression together with the region from −385 to −274 bp.

### OsDof25 recognises the *OsC4PPDK* promoter through a Dof binding motif

**Fig. 4:**
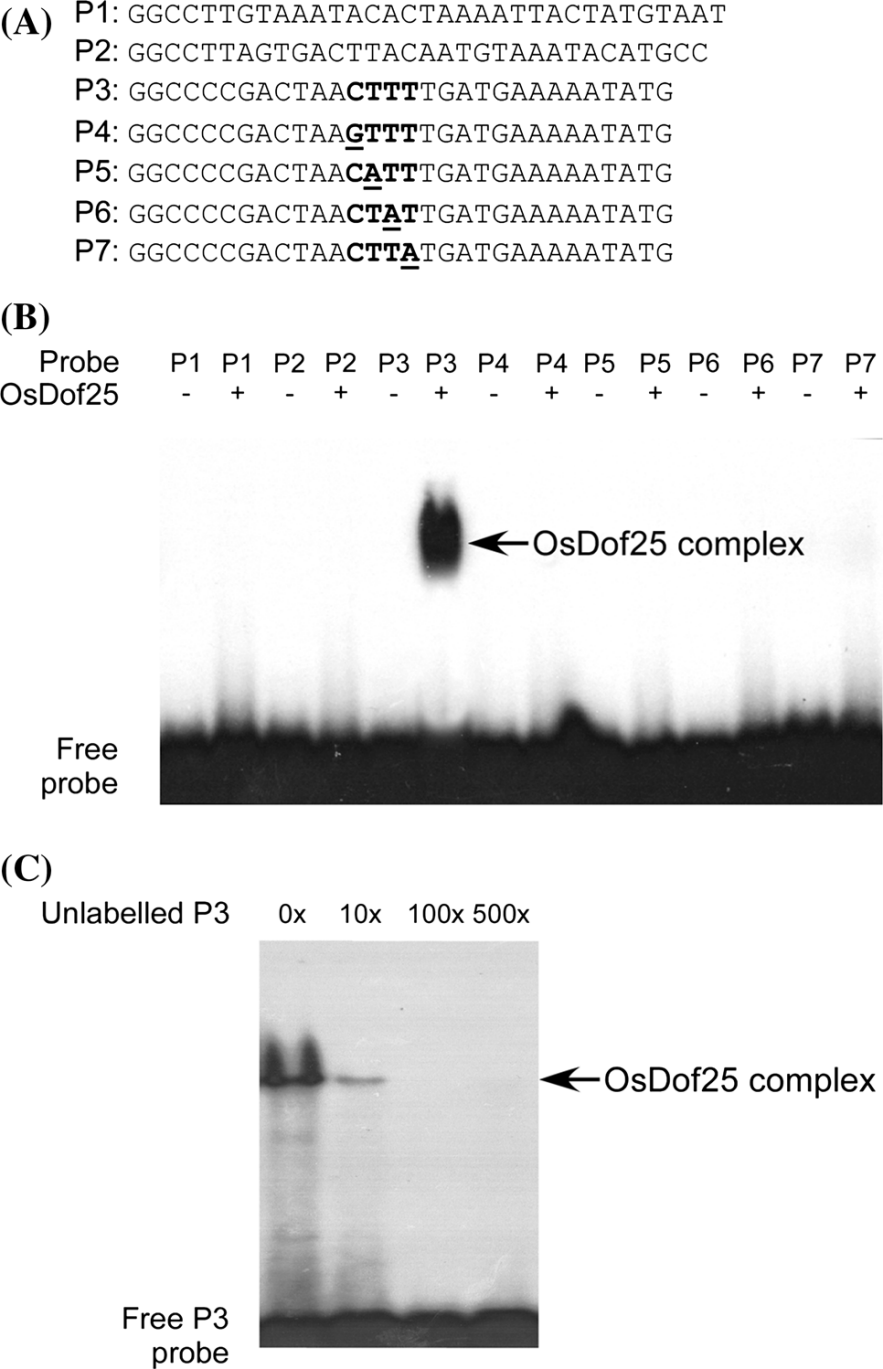
Electrophoretic mobility shift assays (EMSA) of recombinant OsDof25 proteins with oligonucleotides derived from *OsC4PPDK* promoter containing the CTTT motif. **a** Overview of the probes used in EMSAs. P1 and P2 represent two wild type probes without the putative Dof binding site in the *OsC4PPDK* promoter. The wild type probe with the putative Dof binding motif CTTT (P3) and its mutants are also shown. The CTTT motif is shown by bold letters, and its mutants are shown by underlines. **b** The ^32^P-labeled DNA probes were incubated without (−) or with (+) recombinant GST-OsDof25 fusion protein prior to loading on native polyacrylamide gels. **c** OsDof25 protein binds to probe P3 derived from the *OsC4PPDK* promoter. Competitor fragments were added in molar excess as indicated. The positions of the DNA–protein complexes in **b** and **c** are indicated by *arrowhead* and at the *bottom* the free probes are indicated

The transient experiments assays indicated that OsDof25 is able to activate the *OsC4PPDK* promoter through a 111 bp fragment (−385 to −274) containing a putative Dof protein binding site. To confirm whether OsDof25 regulates the *OsC4PPDK* promoter by interacting directly with this specific sequence element, a series of electro mobility shift assays (EMSAs) were conducted. For this, OsDof25 was expressed and purified from *E. coli* as recombinant protein. Next, three different parts of the fragment from −385 to −274 of *OsC4PPDK* promoter with or without the CTTT motif were used as probes (Table S2). In order to determine the specificity of the interaction of OsDof25 protein with the Dof binding motif, a set of four different mutant oligonucleotides was designed and used as EMSA probes (Fig. 4a; Table S2). The EMSA assay of OsDof25 protein with wild type probe P3 produced a distinct complex (Fig. 4b) which could be competed away with a range of unlabelled P3 oligonucleotide (Fig. 4c). On the other hand, when OsDof25 protein was incubated with mutant probes P4, P5, P6 and P7 or two different wild type probes derived from the *OsC4PPDK* promoter (stretching from −385 to −274) without a Dof binding site, than the protein-DNA complex did not appear (Fig. 4b). In conclusion, the EMSAs confirmed that OsDof25 is indeed able to interact specifically with the *OsC4PPDK* promoter through the Dof binding site at position −291 bp.

**Fig. 5:**
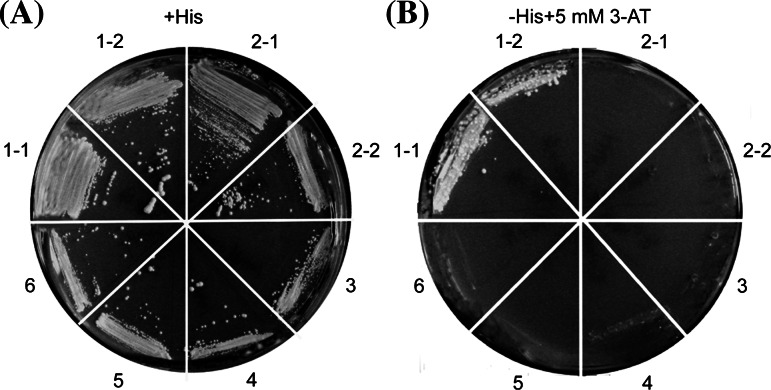
Effects of *OsDof25* overexpression on *OsC4PPDK* promoter-*HIS3* reporter constructs in yeast one-hybrid assays. Reporter constructs ProOsC4PPDK-WT::HIS3, ProOsC4PPDK-MU::HIS3 and an control *HIS3* construct from pINT1-HIS3NB were integrated into the genome of yeast strain Y187 and the resulting strains Y187:ProOsC4PPDK-WT, Y187:ProOsC4PPDK-MU and YPO101 were transformed with pACTIIa-OsDof25 or pACTIIa as empty vector control. Transformed strains were streaked on medium containing histidine (**a**) or on medium without histidine but with 5 mM 3-AT (**b**). Construct pACTIIa-OsDof25 in Y187:ProOsC4PPDK-WT (sectors 1-1, 1-2), pACTIIa-OsDof25 in Y187:ProOsC4PPDK-MU (sectors 2-1, 2-2), pACTIIa-OsDof25 in YPO101 (sector 3), pACTIIa in Y187: ProOsC4PPDK-WT (sector 4), pACTIIa in Y187:ProOsC4PPDK-MU (sector 5), pACTIIa in YPO101 (sector 6). pACTIIa-OsDof25 shows growth on histidine-lacking medium when grown in a strain with construct ProOsC4PPDK-WT::HIS3, but not with ProOsC4PPDK-MU::HIS3 in which the *OsC4PPDK* promoter is mutated or in the control strain YPO101 that contains a control *HIS3* gene preceded by a minimal TATA box-containing promoter

Furthermore, we also studied the interaction between OsDof25 and the *OsC4PPDK* promoter using a yeast one-hybrid system. For this, probes P3 and the mutant P6 (Table S2), were inserted upstream of the reporter gene *HIS3* in the integrative vector pINT1-HIS3NB (Meijer et al. 1998; Ouwerkerk and Meijer 2001) which was transformed into yeast resulting in strains Y187:ProOsC4PPDK-WT and Y187:ProOsC4PPDK-MU. *OsDof25* was expressed in yeast using a GAL4 AD vector. Because of the fusion with the GAL4 Activation Domain (AD), the resulting vector (pACTIIa-OsDof25) can be used for detection of DNA binding of OsDof25 protein to a target sequence without OsDof25 having its own activation domain. Detection of such interaction is via activation of a *HIS3* reporter gene on yeast cells growing on a minimal medium lacking histidine but containing a minimal concentration (5 mM) of 3-AT which is a competitive inhibitor of His3p activity. Figure 5a, shows the growth results of all four combinations of effector and reporters on histidine-containing medium as control to show that none of the effectors has any negative effects on growth. As shown in Fig. 5b, the combination of pACTIIa-OsDof25 and the reporter preceded by the *OsC4PPDK* promoter fragment grew well on minimal medium lacking histidine (Fig. 5b) whereas pACTIIa as empty control did not result in any growth. However, the strain with mutant fragment P6 did not grow on the same minimal medium in combination with either pACTIIa-OsDof25 or pACTIIa (Fig. 5b). Thus, the results from the yeast one-hybrid system show that OsDof25 indeed recognizes the −285 to −274 fragment of *OsC4PPDK* promoter which has a putative Dof binding motif. The specificity of OsDof25 is demonstrated by the lack of activation of a mutant fragment where a sequence CTTT was converted into CTAT.

**Fig. 6:**
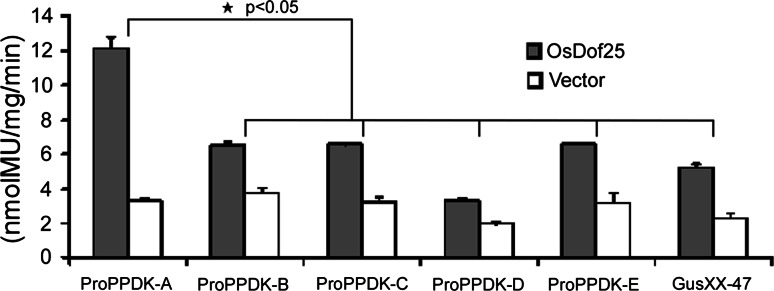
Interaction of OsDof25 with wild type and mutant fragments (−385 to −274) from the *OsC4PPDK* promoter. Effects of OsDof25 overexpression on regulation of *OsC4PPDK* promoter GUS constructs. The GUS reporter construct ProPPDK-A::GUS bears the wild type fragment (−384 to −274) from the *OsC4PPDK* promoter containing motif CTTT. In constructs ProPPDK-B::GUS, ProPPDK-C::GUS, ProPPDK-D::GUS and ProPPDK-E::GUS the wild type motif CTTT is mutated into GTTT, CATT, CTAT and CTTA, respectively. Plasmid pGusXX-47 was used as a negative control for the reporter. The reporter plasmids were co-transformed with Pro35S::OsDof25, or empty effector pRT100. Relative GUS activities were normalised for total protein. The *bar graphs* are based on the mean values of three independent transformations of each construct combination and *error bars* represent the standard deviation (SD) of biological replicates

Initially, a loss-of-function approach identified a 111 bp fragment in the *OsC4PPDK* promoter which is important for regulation by OsDof25. Further EMSAs and yeast one-hybrid studies confirmed specific binding of OsDof25 with a CTTT sequence in this fragment. In order to further confirm the interaction of OsDof25 protein with this fragment *in planta* another series of GUS reporter plasmids were made which encompassed a series of five constructs (ProPPDK-A::GUS, ProPPDK-B::GUS, ProPPDK-C::GUS, ProPPDK-D::GUS and ProPPDK-E::GUS) for a gain-of-function approach in combination with transient expression in protoplasts. These constructs were based on the 111 bp fragment from −385 to −274 bp and represented the wild type fragment and four mutants in the CTTT motif (Table S2). As shown in Fig. 6, the wild type fragment (construct ProPPDK-A::GUS) was activated by OsDof25 by fivefold, but when either of the four mutant fragments are used, the ratio of induction drops down to twofold as the empty constructs. Together, the results show that OsDof25 can interact in vitro and in vivo specifically with a Dof binding motif close to the ORF of *OsC4PPDK* and may play an important role *in planta* regulation too. Since OsDof24 is the closest homologue in the phylogeny tree with OsDof25 (Fig. 2), we also tested if this gene is able to activate the same *OsC4PPDK* promoter constructs as used in the loss-of-function and gain-of-function analyses. As shown in Fig. S1, the effects of OsDof24 expression in the protoplast system are essentially the same as with OsDof25 (Figs. 3, 6).

### Sub-cellular localisation of OsDof25 transcription factor in rice protoplasts

**Fig. 7:**
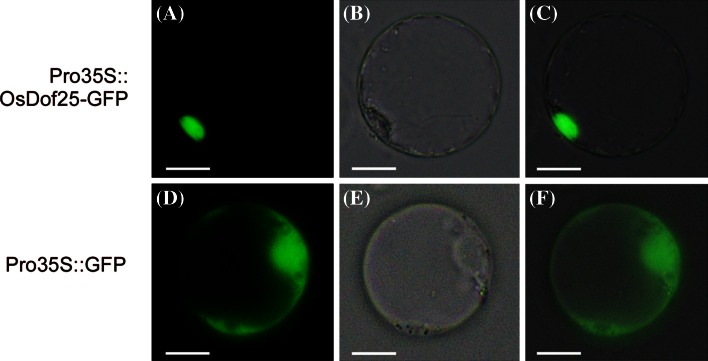
Sub-cellular localisation of OsDof25 protein in rice seedling protoplasts. Rice protoplasts were transiently transformed with Pro35S::OsDof25-GFP (**a, b, c**) and Pro35S::GFP (**d, e, f**). After overnight incubation, cells were observed with fluorescence (**a, d**) and bright field (**b, e**) microscopy. A merged picture of **a** and **b** and **d** and **e**, respectively, were shown in panels **c** and **f**. *Scale bar* represent 2 µm

The subcellular localisation of OsDof25 protein was studied using transient expression of a GFP-tagged OsDof25 fusion protein in rice protoplasts. For this, the ORF of *OsDof25* was fused in frame to the N-terminus of the *GFP* gene to generate plasmid Pro35S::OsDof25-GFP. The empty plasmid pTH2 (Pro35::GFP) was used as a control (Chiu et al. 1996). Fluorescence was detected using confocal laser scanning microscopy (CSLM). As shown in Fig. 7, GFP-tagged OsDof25 was specifically localised in the cell nucleus, whereas the control GFP protein was localised in both cytoplasm and the nucleus. This experiment confirmed that the OsDof25 protein is a nuclear-localised protein which is consistent with a function as transcription factor.

### Expression profiling of *OsDof25* and *OsC4PPDK*

**Fig. 8:**
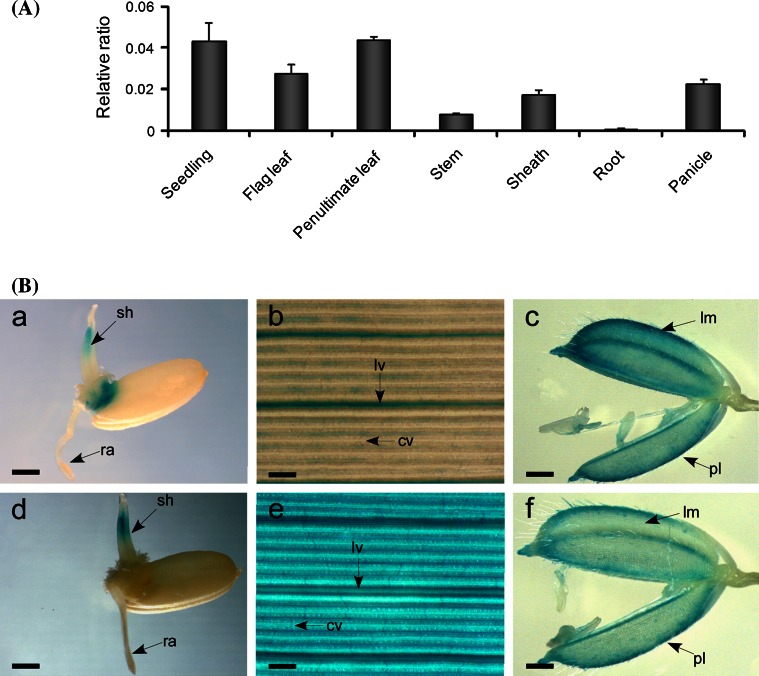
Characterisation of the expression profile of *OsDof25* and *OsC4PPDK.* **a** Expression profile of *OsDof25* in different tissues which are from left to right, 2 week old seedlings, 10 DAF (days after flowering) flag leaf, penultimate leaf, stem, sheath, panicle and root. The *Ubi* gene was used as control for normalisation of cDNA quantity. *Bars* represent means standard error (n = 3 independent qPCRs). **b** Histochemical localisation of *OsDof25* (*a*–*c*) and *OsC4PPDK* (*d*–*f*) promoter-GUS expression in transgenic rice. *a*–*d* Two days old-germinating seeds, *b*–*e* flag leaf, *c*–*f* immature spikelets. *Scale bars* represent 1 mm (*a, d*), 100 µm (*b, e*) and 500 µm (*c, f*) respectively. *cv* commissural vein, *lv* longitudinal vein, *lm* lemma, *pl* palea, *ra* radicle, *sh* shoot

The expression profile of *OsDof25* was studied using qPCR and promoter GUS plants. The qPCR assays showed that the expression of *OsDof25* is not strictly tissue-specific, but has highest expression in penultimate leaves at 10 days after flowering (DAF) and 2 week old seedlings, followed by flag leaves at 10 DAF, panicle, leaf sheath and stem and lowest expression in roots (Fig. 8a). To further study the expression pattern of *OsDof25* and the possible overlap with *OsC4PPDK* in more detail, we generated ProOsDof25::GUS and ProOsC4PPDK::GUS transgenic lines. For both *OsDof25* and *OsC4PPDK* promoters, the GUS signal was observed in leaves and florets as well as in germinating seeds but there was no GUS signal detected in radicles which is in accordance to the qPCR data for *OsDof25*. For *OsC4PPDK*, GUS activity was also detected in immature seeds, where no expression for *OsDof25* could be detected (Fig. 8b).

Taken together, the results demonstrated that *OsDof25* is expressed in different tissues and at different developmental stages. The GUS signals detected in ProOsDof25::GUS rice are in accordance with the expression profiles of *OsDof25* determined by qPCR and overlap with those of *OsC4PPDK*, indicating that OsDof25 may play an important role in rice growth and development besides playing a key role on the transcription of *OsC4PPDK* but we cannot rule out entirely different functions.

### Functions of OsDof25 in regulation of *OsC4PPDK* expression in rice

The regulation of *OsC4PPDK* by OsDof25 was further studied using loss-of-function and gain-of-function approaches in transgenic rice. To determine whether OsDof25 is required for expression of the *OsC4PPDK* gene, we tried to down-regulate *OsDof25* expression using RNA interference (RNAi) with the pHANNIBAL system (Wesley et al. 2001). For this, a binary vector was made carrying an inverted repeat of a specific part of the *OsDof25* and used in rice transformation. Five independent single copy transgenic lines were identified using Southern blotting analysis (Fig. S2a) and used in further analysis. Among the five lines transformed with the *OsDof25* silencing construct, two lines, numbers #5 and #30, showed a decrease in *OsDof25* mRNA level as shown by qPCR analysis (Fig. S2b) but no obvious phenotype was visible. Next, the T_1_ generation of RNAi-*OsDof25* line #5 and line #30 were analysed in more detail for the effect on *OsC4PPDK* expression. Both RNAi lines showed a reduction in the level of *OsC4PPDK* mRNA compared to the control lines which were non-transgenic lines separated from the T_0_ (Fig. S2c).

The RNAi experiments demonstrated that OsDof25 plays a role in the expression of *OsC4PPDK*. To determine whether an elevated level of *OsDof25* expression is sufficient for activating the expression of *OsC4PPDK*, transgenic rice plants were made, which were transformed with a binary vector carrying the *OsDof25* gene driven by the constitutive *GOS2* promoter (De Pater et al. 1992; Ouwerkerk et al. 2001). Three independent single copy number lines were selected using Southern blotting (Fig. S3a) and qPCR assays showed that all three transgenic lines had significantly elevated *OsDof25* mRNA levels compared to the control lines (Fig. S3b). These lines did not show obvious differences in phenotype but analysis of the expression level of *OsC4PPDK* by qPCR showed significant up-regulation (Fig. S3c). Since *OsC4PPDK* is important in photosynthesis we also checked photosynthetic capacity in the *OsDof25* overexpressors and RNAi lines but we did not observe a significant and large difference with the wild type lines (Fig. S4). Taken together, both loss-of-function and gain-of-function studies with OsDof25 showed a clear and opposite effect as activator of *OsC4PPDK* expression which confirmed the results obtained from the EMSA, yeast one-hybrid assay and transient expression experiments in rice protoplasts where we showed binding of OsDof25 with the *OsC4PPDK* promoter.

## Discussion

In a comparative study between *Dof* genes from rice and maize we identified the paralogous pair *OsDof24* and *OsDof25* as orthologues of *ZmDof1*. Little is known about the precise functions of Dof genes although some members were characterised in rice (Gaur et al. 2011; Nie et al. 2013), Arabidopsis, Sorghum (Kushwaha et al. 2011), tomato (Cao et al. 2013), *Brachypodium* (Hernando-Amado et al. 2012) and soybean (Guo and Qiu 2013). In rice for *OsDof12* (Li et al. 2009), a function in flowering time was described in detail. *OsDof24* is also named *OsDof25* because of a different nomenclature (Santos et al. 2012) and has been implicated in regulation of genes involved in carbon and nitrogen metabolism but this is a different gene than the *OsDof25* gene described here. Other *Dof* genes such as *BPBF* from barley (Diaz et al. 2002) and several genes from *Brachypodium* (Hernando-Amado et al. 2012) are expressed during grain development and seem to play a role in this process but this seems not be the case for *OsDof25* showing that although *Dof* genes bind similar *cis*-acting elements, they may still have functions in different tissues and biological processes. More than half of the Dof members in Arabidopsis are expressed in vascular tissues and may have roles in vascular development and functioning such as short or long-distance signaling (Le Hir and Bellini 2013). We found that *OsDof25* was also expressed in vascular tissue, which suggests that this gene could also play a role in development of vascular tissues or in regulation of genes involved in vascular loading or unloading or other processes acting in this tissue.

In maize, *Dof1* is known to have functions in regulation of *C4PPDK* (Yanagisawa 2000) which plays an important role in C4 photosynthesis (Chollet et al. 1996). Since *OsDof25* is a close homologue of *ZmDof1* which is involved in regulation of *PPDK*, we conducted several studies to check whether this gene–gene interaction is conserved in rice. In order to understand the expression of the mechanism by which OsDof25 potentially could regulate *OsC4PPDK*, we delineated the *OsC4PPDK* promoter and identified a minimal fragment with only one Dof binding site that still gave activation by OsDof25 in a transient expression analysis. Functionality of this site in regulating *OsC4PPDK* expression was proven by mutation analysis that showed reduced binding in in vitro and in yeast one-hybrid experiments which was next confirmed with the transient protoplast experiments. Important data for a role of OsDof25 in regulation of *OsC4PPDK* came from a loss-of-function and gain-of-function analysis where down-regulated or up-regulated *OsDof25* expression correlated with lower or higher *OsC4PPDK* expression respectively. Furthermore, no adverse phenotypical effects were seen in these transgenics which also increases the usefulness in biotechnological applications. Because OsDof24 is closest to OsDof25, we also did some analyses on this gene and found that it is also able to regulate the *OsC4PPDK* promoter in rice protoplasts in the same way as OsDof25 did. However, although we did not check for *OsC4PPDK* expression in *OsDof24* overexpression rice transgenics, we came to the conclusion that these genes will have different downstream targets since these plants showed an obvious delay in flowering time and strong effects on flowering time genes were observed (Yu et al., unpublished results).

The overlap in expression patterns of the *OsDof25* and *OsC4PPDK* genes in green tissues is confirmed by the promoter GUS analyses and is in accordance with the transgenic experiments that show that OsDof25 is a key activating regulator of *OsC4PPDK.* Future experiments will have to show whether this is also the case for other photosynthesis genes in either the C3 or C4 pathways. Since *OsDof25* is also expressed in non-photosynthetically active tissues, this activator will likely also be involved in completely other pathways. Although rice is not a typical C4 plant like maize or Sorghum since it does not have the typical Kranz anatomy to capture CO_2_ nor does rice have the accompanying photosynthetic capacity, C4 photosynthesis genes are present (Bao et al. 2005; Song et al. 2010). Chinese hybrid rice combinations can show a slightly elevated photosynthetic capacity and it has been speculated that enhanced activity of C4 photosynthesis enzymes is responsible for this phenomenon (Bao et al. 2005; Song et al. 2010) but it needs still to be investigated if the *Dof* genes such as *OsDof25* are involved in any differential expression of downstream targets. We also observed that the photosynthetic capacity of *OsDof25* overexpression and RNAi lines was not different from the controls. However, transgenic overexpression studies with the maize *PEPC* and *PPDK* genes in rice and wheat showed a clear effect on photosynthetic capacity and certain yield components such as increased grain yield also because of increased tillering (Ku et al. 2001; Zhang et al. 2014). However, these experiments are quite different than our set-up where *OsC4PPDK* was higher expressed due to overexpression of an upstream regulator which may mean that *OsC4PPDK* expression is not higher in all cell types.

To summarise, in this study we confirmed an *in planta* interaction between the *cis*-element of the *OsC4PPDK* promoter and OsDof25 and we confirmed that this gene is an activator of *OsC4PPDK*. However, since photosynthetic capacity is not increased due to *OsDof25* over-expression, likely also other genes will be required to achieve this and increase yield in this way.

## Electronic supplementary material

Supplementary material 1 (PDF 864 kb)

## Abbreviations

Dof: DNA binding with one finger
GUS: β-Glucuronidase
MS: Murashige–Skoog
*OsC4PPDK*: Pyruvate orthophosphate dikinase
