# Supplementary material for: Functions of OsDof25 in regulation of OsC4PPDK
Source: Plant Mol Biol. 2015 Sep 3;89(3):229–42. doi: 10.1007/s11103-015-0357-3 (PMC4579267; doi:10.1007/s11103-015-0357-3)
Supplement: Supplementary file 1 — Supplementary material 1 (PDF 864 kb) [file 11103_2015_357_MOESM1_ESM.pdf]

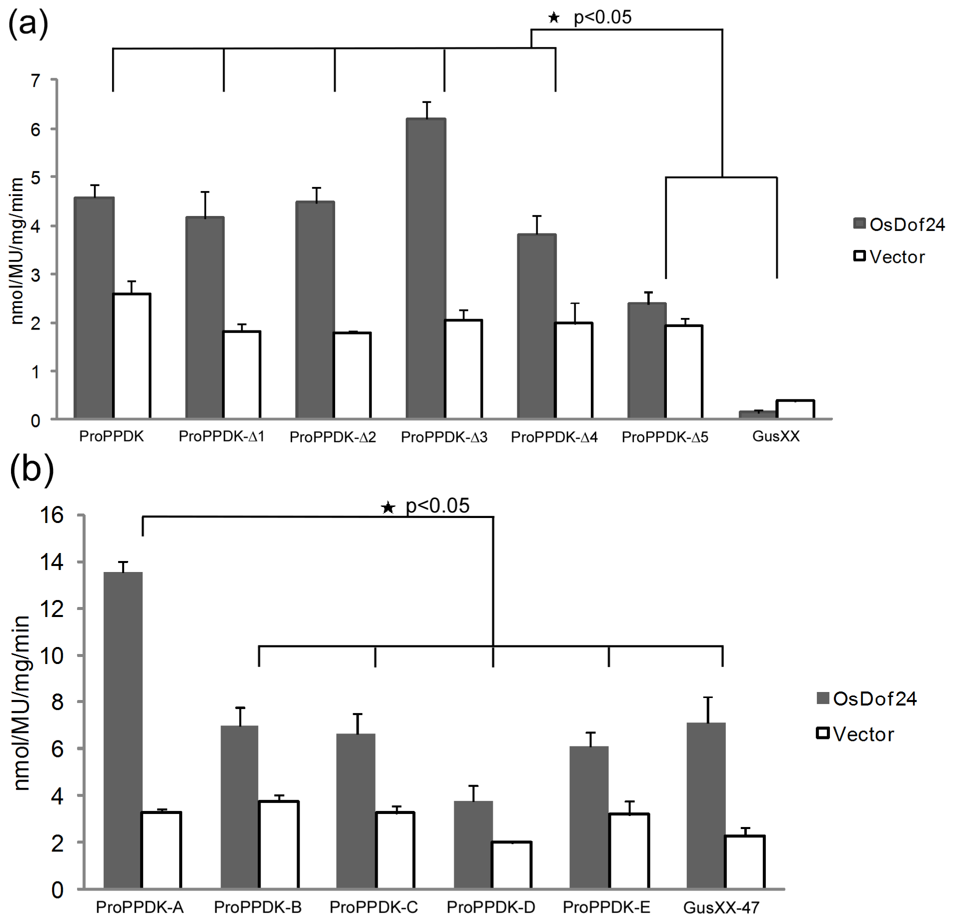

Fig S1. Interactions of OsDof24 with the *OsC4PPDK* promoter in rice protoplasts

(**a**) **Loss-of-function analysis of the *OsC4PPDK* promoter** Effects of OsDof24 overexpression and the mapping of OsDof24-binding fragments on the *OsC4PPDK* promoter were tested using an overexpression construct Pro35S::OsDof24 which was co-transformed into rice protoplasts with a series of *OsC4PPDK* promoter deletion GUS constructs. GUS activities of co-transformation with Pro35S::OsDof24 are indicated in black and columns representing empty effector plasmids are blank.

(**b**) **Gain-of-function analysis of OsDof25 with wild type and mutant fragments (-385 to -274) from the *OsC4PPDK* promoter** The GUS reporter construct ProPPDK-A::GUS bears the wild type fragment (-384 to -274) from the *OsC4PPDK* promoter containing motif CTTT. In constructs ProPPDK-B::GUS, ProPPDK-C::GUS, ProPPDK-D::GUS and ProPPDK-E::GUS the wild type motif CTTT is mutated into GTTT, CATT, CTAT and CTTA, respectively. Plasmid pGusXX-47 was used as a negative control for the reporter. In both panels, the reporter plasmids were co-transformed with Pro35S::OsDof24, or empty effector pRT100. Relative GUS activities were normalised for total protein. The bar graphs are based on the mean values of three independent transformations of each construct combination and error bars represent the standard deviation (SD) of biological replicates. The data were analysed using ANOVA followed by Bonferroni corrections. Asterisks indicate significant differences ($p<0.05$) compared with the untransformed controls.

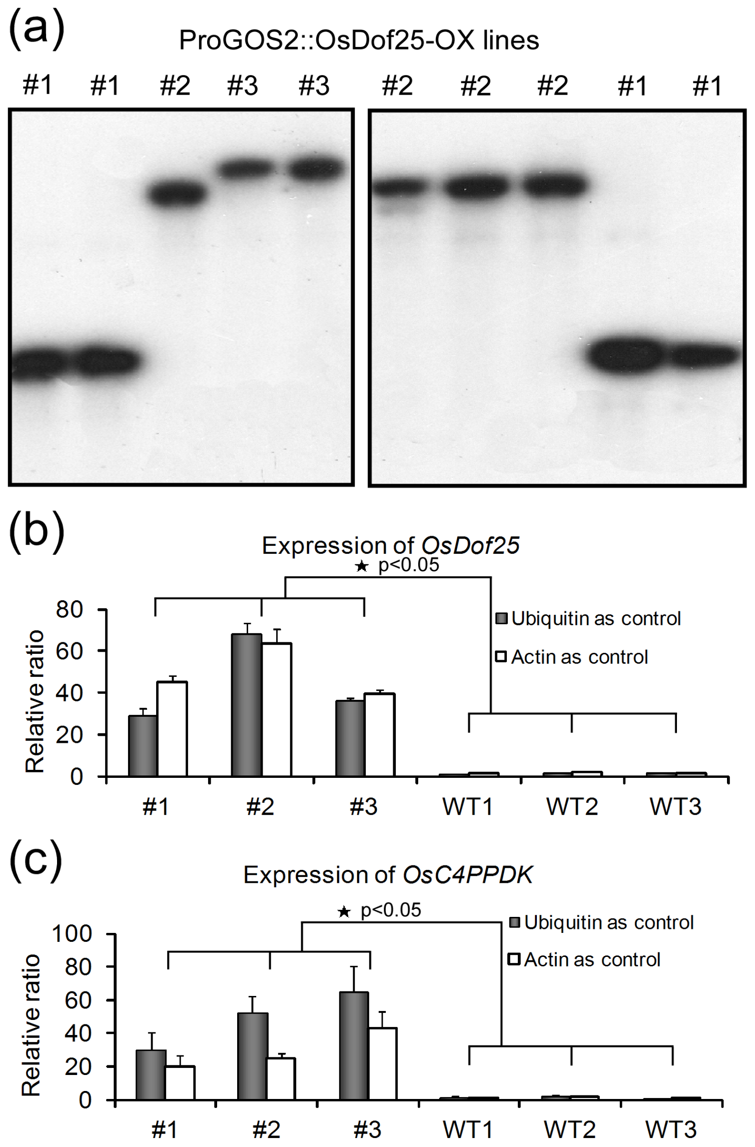

Fig S2. Molecular analysis of *OsDof25* RNAi plants

(**a**) Southern blotting results showing the T-DNA insertion copy number. The *hpt* gene was used as probe. (**b**) Analysis of *OsDof25* expression in T_1 *OsDof25* RNAi plants (lines #5 and #30) and control plants, azygous plants separated from the T_0 (WT). (**c**) Analysis of *OsC4PPDK* expression in T_1 *OsDof25* RNAi plants (lines #5 and #30) and control plants, which are azygous plants separated from the T_0 (WT). *Ubiquitin* and *Actin* genes were used for equilibration of cDNA quantity in qPCR experiments. Bars represent means standard error (n=3 independent qPCRs). The bargraphs are based on the mean values of three independent transformations of each construct combination and error bars represent the standard deviation (SE) of biological replicates. The data were analysed using ANOVA followed by Bonferroni corrections. Asterisks indicate significant differences ($p<0.05$) compared with the untransformed controls.

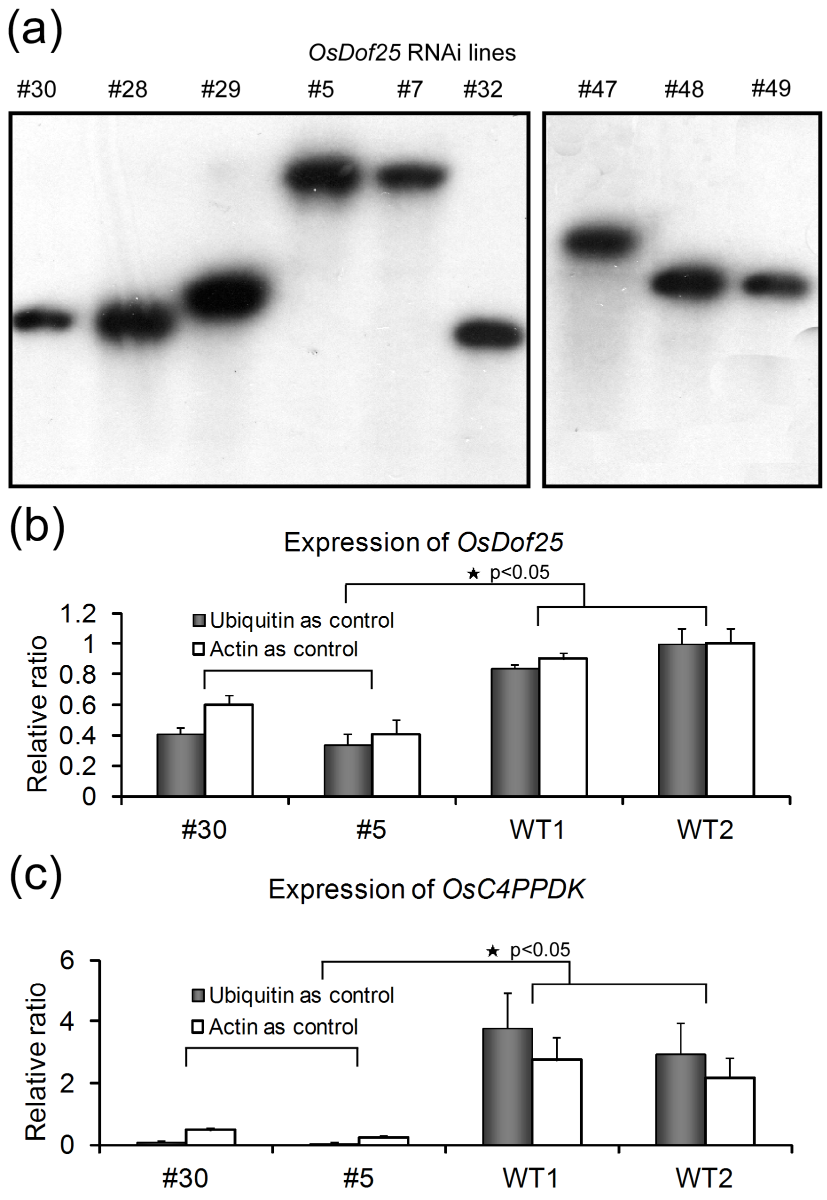

Fig S3. Molecular analysis of transgenic plants overexpressing *OsDof25*

(**a**) Southern blotting results showing the T-DNA insertion copy number. The *hpt* gene was used as probe. (**b**) Expression analysis of *OsDof25* in T_1 ProGOS2::OsDof25 plants (lines #1, #2 and #3) and azygous controls segregated from the T_0 (WT). (**c**) Expression analysis of *OsC4PPDK* in ProGOS2::OsDof25 T_1 plants (lines #1, #2 and #3) and azygous control plants, segregated from the T_0 (WT). *Ubiquitin* and *Actin* genes were used for equilibration of cDNA quantity in qPCR experiments. The bar graphs are based on the mean values of three independent transformations of each construct combination and error bars represent the standard deviation (SD) of biological replicates. The data were analysed using ANOVA followed by Bonferroni corrections. Asterisks indicate significant differences ($p<0.05$) compared with the untransformed controls.

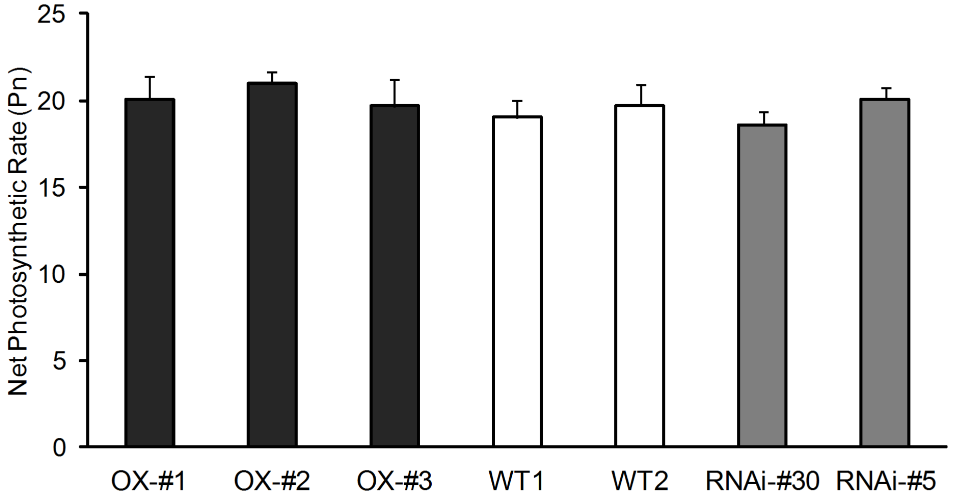

Fig S4. Analysis of the photosynthetic capacity in OsDof25-OX and RNAi lines. Photosynthetic capacity was determined under field conditions at day 5 after flowering on flag leaves from OsDof25 overexpression and RNAi plants and compared to control plants. The bar graphs are based on the mean values of three independent plants of each line and error bars represent the standard deviation (SD) of biological replicates.

Table S1. Overview of the Dof transcription factor family as identified in japonica rice cultivar Nipponbare (Lijavetzky et al. 2003; Yang et al. 2006; Jin et al. 2014). Indicated are the gene names, locus codes in TIGR, chromosomal coordinates in Mb and GenBank Accession codes for BAC or PAC clones.

Gene code	Japonica BAC/PAC clone	Position on chromosome (cm)	Locus code	Chromosome number
OsDof1	P0505D12	37.8	LOC_Os01g64590	1
OsDof2	P0453A06	8.9	LOC_Os01g15900	1
OsDof3	P0671B11	5	LOC_Os01g09720	1
OsDof4	P0038F12	9.7	LOC_Os01g17000	1
OsDof5	P0007F06	28	LOC_Os01g48290	1
OsDof6	B1131G08	32.1	LOC_Os01g55340	1
OsDof7	P0680A05	29.2	LOC_Os02g47810	2
OsDof8	B1121A12	30.2	LOC_Os02g49440	2
OsDof9	P0657H12	27.4	LOC_Os02g45200	2
OsDof10	OSJNBa0009N02	8.6	LOC_Os02g15350	2
OsDof11	OSJNBa0010D22	21.5	LOC_Os03g38870	3
OsDof12	OSJNBa0091P11	3.7	LOC_Os03g07360	3
OsDof13	OSJNBa0063J18	23.4	LOC_Os03g42200	3
OsDof14	OSJNBb0014A21	9.3	LOC_Os03g16850	3
OsDof15	OSJNBa0079B15	31.6	LOC_Os03g55610	3
OsDof16	OJ1754_E06	34.4	LOC_Os03g60630	3
OsDof17	OSJNBa0064G10	34.4	LOC_Os04g58190	4
OsDof18	OSJNB0005N02	28.3	LOC_Os04g47990	4
OsDof19	P0016H04	0.6	LOC_Os05g02150	5
OsDof20	P0491D10	10.1	LOC_Os06g17410	6
OsDof21	P0407H12	7.6	LOC_Os07g13260	7
OsDof22	OJ1163_G04	19.3	LOC_Os07g32510	7
OsDof23	OSJNBa0060O17	29	LOC_Os07g48570	7
OsDof24	P0605H02	24.1	LOC_Os08g38220	8
OsDof25	P0556A05	18.2	LOC_Os09g29960	9
OsDof26	OSJNBa0060A14	13.5	LOC_Os10g26620	10
OsDof27	OSJNBa0066I08	18.5	LOC_Os10g35300	10
OsDof28	OSJNBa0016C14	23.4	LOC_Os12g38200	12
OsDof29	B1110B01	21.4	LOC_Os05g36900	5
OsDof30	OSJNBa0044E20	24.7	LOC_Os12g39990	12

Table S2. List of oligonucleotides used in EMSA and yeast experiments. Overview of sequences of both wild type and mutant fragments of the *OsC4PPDK* promoter, which were used in EMSA and yeast experiments. The putative Dof protein binding motifs, CTTT, are labeled in bold and the mutant series are indicated by underlines.

Name	Sequence (5' to 3')
P1 up	GGCCTTGTAAATACACTAAAATTACATATGTAAT
P1 down	CTAGATTACATATGTAATTTTAGTGTATTTACAA
P2up	GGCCTTAGTGACTTACAATGTAAATACATGCC
P2 down	CTAGGGCATGTATTTACATTGTAAGTCACTAA
P3up	GGCCCCGACTAA**CTTT**TGATGAAAAATATG
P3 down	CTAGCATATTTTTCATCAAAAGTTAGTCGG
P4 up	GGCCCCGACTAA**GTTT**TGATGAAAAATATG
P4 down	CTAGCATATTTTTCATCAAAACTTAGTCGG
P5 up	GGCCCCGACTAA**CATT**TGATGAAAAATATG
P5down	CTAGCATATTTTTCATCAAATGTTAGTCGG
P6 up	GGCCCCGACTAA**CTAT**TGATGAAAAATATG
P6 down	CTAGCATATTTTTCATCAATAGTTAGTCGG
P7 up	GGCCCCGACTAA**CTTA**TGATGAAAAATATG
P7 down	CTAGCATATTTTTCATCATAAGTTAGTCGG

Table S3. List of oligonucleotides used for the construction of GUS reporter plasmids, and used in PCR. Putative Dof binding motifs in the *OsC4PPDK* promoter are indicated in bold and mutant sites are indicated by underlining.

Name	Sequences (from 5' to 3')
ProPPDK-F	ATAAGAATGCGGCCGCGTACTTACATATATAATTTTG
ProPPDK-R-A	GGACTAGTCCATATTTTTCATCA**AAAG**TTAG
ProPPDK-R-B	GGACTAGTCCATATTTTTCATCA**AAAC**TTAG
ProPPDK-R-C	GGACTAGTCCATATTTTTCATCA**AATG**TTAG
ProPPDK-R-D	GGACTAGTCCATATTTTTCATCA**ATAG**TTAG
ProPPDK-R-E	GGACTAGTCCATATTTTTCATCA**TAAG**TTAG
ProOsC4PPDK-F	GGAATTCCTGGTTTCCACGGCGTGCAACGGTGGC
ProOsC4PPDK-R	CCATGGCCTGGCCCTGATCGATC
OsDof25-F1	CGGGATCCATGCAGGAGGCGGGGCGAC
OsDof25-R1	GGAATTCTCCATGGCAGGTTAAGGAACAGTG
OsDof25-F2	CCGCTCGAGATGCAGGAGGCGGGGCGAC
OsDof25-R2	CATGCCATGGCTGGCAGGTTAAGGAACAG
OsDof25-F3	TAATACGACTCACTATAGGGCACAACCAAGAAAGCCCCGG
OsDof25-R3	TGGCAGGTTAAGGAACAGTGATGG
OsDof25-F4	TAATACGACTCACTATAGGGTGGCAGGTTAAGGAACAGTGATGG
OsDof25-R4	CACAACCAAGAAAGCCCCGG
OsDof25-F5	CATGCCATGGAGGAGGCGGGGCGACGG
OsDof25-R5	CGGGATCCTCATGGCAGGTTAAGGAAC
OsDof25-F6	CCATCGATATCGGTGTCGGCGAACAC
OsDof25-R6	CGGGATCCCACCACCGCCGACGCTG
OsDof25-F7	CCGCTCGAGCACCACCGCCGACGCTG
OsDof25-R7	GGGGTACCATCGGTGTCGGCGAACAC
ProOsDof25-F	CCTGAGAACTCAGACGCTATGACGAAAC
ProOsDof25-R	CCATGGCGCGCCCGCAGAAAAGCTCGGACGAAC

Table S4. List of oligonucleotides used for qPCR

Name	Sequences (from 5' to 3')
*Actin*F	GACCCAGATCATGTTTGAGACC
*Actin*R	CATCACCAGAGTCCAACACAATAC
*Ubiquitin*F	AGCAGAAGCACAAGCACAAG
*Ubiquitin*R	AAGCCTGCTGGTTGTAGACG
OsDof25-F	AAGGAGCACCTGGACACGA
OsDof25-R	GGCAGGTTAAGGAACAGTGATGGA
OsC4PPDK-F	GATCCGTCGTGGCGCAGA
OsC4PPDK-R	CTGAGAGGCGAGCAATGC
